# Diabetes-related molecular signatures in infrared spectra of human saliva

**DOI:** 10.1186/1758-5996-2-48

**Published:** 2010-07-14

**Authors:** David A Scott, Diane E Renaud, Sathya Krishnasamy, Pinar Meriç, Nurcan Buduneli, Şvetki Çetinkalp, Kan-Zhi Liu

**Affiliations:** 1 Oral Health and Systemic Disease, University of Louisville, Louisville, S Preston St, Louisville, KY, 40292, USA; 2 Department of Pharmacology and Toxicology, University of Louisville, Louisville, S Preston St, Louisville, KY, 40292, USA; 3 Department of Microbiology and Immunology, University of Louisville, Louisville, S Preston St, Louisville, KY, 40292, USA; 4 Endocrinology, University of Louisville, Louisville, S Jackson St, Louisville, KY, 40292, USA; 5 Department of Periodontology, Ege University, Bornova, İzmir, 35100, Turkey; 6 Metabolic Diseases and Endocrinology, Ege University, Bornova, İzmir, 35100, Turkey; 7 Institute for Biodiagnostics, National Research Council, Ellice Avenue, Winnipeg, MB, R3B 1Y6, Canada; 8 Department of Oral Biology, University of Manitoba, Bannatyne Avenue, Winnipeg, MB, R3E 0W2, Canada

## Abstract

**Background:**

There is an ongoing need for improvements in non-invasive, point-of-care tools for the diagnosis and prognosis of diabetes mellitus. Ideally, such technologies would allow for community screening.

**Methods:**

In this study, we employed infrared spectroscopy as a novel diagnostic tool in the prediction of diabetic status by analyzing the molecular and sub-molecular spectral signatures of saliva collected from subjects with diabetes (*n *= 39) and healthy controls (*n *= 22).

**Results:**

Spectral analysis revealed differences in several major metabolic components - lipid, proteins, glucose, thiocyanate and carboxylate - that clearly demarcate healthy and diseased saliva. The overall accuracy for the diagnosis of diabetes based on infrared spectroscopy was 100% on the training set and 88.2% on the validation set. Therefore, we have established that infrared spectroscopy can be used to generate complex biochemical profiles in saliva and identify several potential diabetes-associated spectral features.

**Conclusions:**

Infrared spectroscopy may represent an appropriate tool with which to identify novel diseases mechanisms, risk factors for diabetic complications and markers of therapeutic efficacy. Further study into the potential utility of infrared spectroscopy as diagnostic and prognostic tool for diabetes is warranted.

## Background

The rapid, unequivocal diagnosis of both type 1 and type 2 diabetes is essential to avoid the onset of complications. Diabetes is a remarkably complex disease, yet diagnosis is determined by measurements of a single biomarker - glucose. The degree of hyperglycemia changes over time and reflects both the severity of the underlying metabolic process and the success of treatment [1]. The American Diabetes Association (ADA) treatment guidelines suggest that preprandial capillary plasma glucose concentrations should be in the range of 90-130 mg/dl (5.0-7.2 mmol/l), but that HbA1c (glycosylated haemoglobin type A1c, < 7%) is the primary target for glycemic control [2]. Other measures such as fructosamine and glycated albumin are available as markers of hyperglycemia, but there have been no definitive ranges established to allow treatment to goal [3,4].

While there are no accepted diagnostic markers for diabetes other than glucose, many molecules have been tested for their diagnostic potential, and several biomarkers have been identified that provide important adjunctive information. For example, ketone (β-hydroxybutyrate) measurement, normally in urine, is useful in diagnosing diabetic ketoacidosis [5]. Albumin excretion into urine is often used to monitor deteriorating renal health [6], with creatinine clearance an additional available tool [7]. Lipid profiling (total cholesterol, triglycerides, HDL and LDL) is recommended for diabetics, with aggressive treatment provided to those with dyslipidemia [2]. Specific auto-antibodies, such as islet cell cytoplasmic, insulin, glutamic acid decarboxylase, and islet cell antigen 512 (IA2/ICA512) autoantigen, combined with other metabolic and genetic markers, are effective for predicting eventual development of type 1 diabetes in otherwise healthy individuals [8]. Autoimmune diagnostics are of particular importance to discriminate between type 1 and type 2 diabetes and for the differential diagnosis of type 1 diabetes when clinical and metabolic criteria alone do not allow definite classification [9]. Advanced glycation end products (AGEs; glycoxidation post-translational modifications of a variety of polypeptides) and advanced lipoxidation end products (ALEs; lipoxygenation post-synthesis modifications of a variety of lipids), which promote inflammation, have also been proposed as diagnostic and prognostic markers [3,10]. The accumulation of AGE products is virtually irreversible; hence AGE formation is likely to impart a long-term effect on the tissues [11,12].

Recent research suggests that specific salivary biomarkers such as glucose, α-amylase, and ghrelin appetite hormone exhibit strong diagnostic potential for diabetes [13-15]. Other potential diabetes-related biomarkers have also been detected in saliva, including immunoglobulins, glycated end products, and other markers of oxidative status, such as myeloperoxidase, salivary peroxidise, and multiple other oxidants [13-18]. Many such biomarkers will exhibit unique signatures in the IR spectrum of saliva.

Infrared (IR) spectroscopy can be employed to monitor all molecules present in saliva rapidly and simultaneously. Briefly, the attenuation of the intensity of a beam of infrared light upon passing through a sample is measured. The intensities of IR spectra provide quantitative information, while the frequencies reveal qualitative characteristics about the nature of the chemical bonds, their structure, and their molecular environment. Thus, an IR spectrum is the sum of all such contributions and represents a molecular fingerprint including those changes to cells, tissues, or fluids that accompany all pathological processes. In the recent decades, IR spectroscopy has demonstrated its strong potential in detecting small and early biochemical changes associated with disease. IR spectroscopy has been successfully adapted, for example, to predict fetal lung maturity [19], diagnose heart disease [20], rheumatoid arthritis [21] and Alzheimer's disease [22], as well as to, e.g., to monitor lipidemia [23] using serum/plasma samples. We expected that diabetes would induce multiple and specific alterations to the molecular profile of saliva and that these pathogenic changes could be readily detected by IR spectroscopy. Indeed, FT-IR spectroscopy has been previously employed to monitor some specific molecules that also represent diabetes-related signals such as early glycation (Amadori) products [24] and glucose [25].Therefore, we set out to monitor diabetes-specific alterations to the molecular composition of saliva from subjects with diabetes and control subjects using this unique optical tool.

## Methods

### Study population

Thirty-nine patients diagnosed as having diabetes for at least one year prior to the study using American Diabetes Association diagnostic criteria and who were consecutively referred to the Department of Metabolic Diseases and Endocrinology at Ege University were recruited by a single clinician (ŞÇ). Type 1 and type 2 diabetes were distinguished by patient history combined with clinical characteristics and by autoantibody testing (anti-GAD, ICA) in cases where medical history and clinical findings were not characteristic. If type 1 and 2 diabetes were not distinguished by the aforementioned criteria, C peptide analysis was employed. Twenty-one systemically healthy control subjects without diabetes were recruited from patients seeking dental treatment at the School of Dentistry, Ege University. There were no exclusion criteria. Written, informed consent was obtained from all subjects, as approved by the local ethics committee in Turkey. The demographics and clinical characteristics of total population are presented in Table 1. There were no statistically significant differences between subjects and controls in gender or smoking status, although the mean age of the diabetes group was slightly higher.

**Table 1:** Demographics and clinical characteristics of study population

	Subjects (n = 39)	Controls (n = 23)
**Age (years, *s.d*.)**	46.3, *14.9*	38.2, 13.3*
**Gender (*% F*)**	62	61
**Smokers (%)**	59	61
**HbA1c (%, *s.d*.)**	7.4, *2.1*	ND
**Blood glucose (mg/dL, *s.d*.)**	149.6, *80.6*	ND
**Cholesterol (mg/dL, *s.d*.)**	206.9,*101.8*	ND
**HDL, (mg/dL, *s.d*.)**	48.0,*14.1*	ND
**LDL (mg/dL, *s.d*.)**	123.7, *54.4*	ND

* ***p ***= 0.044; ND: Not determined.

### Saliva sampling

Saliva samples were obtained in the morning following an overnight fast during which subjects were requested not to drink (except water) or chew gum. Unstimulated whole saliva samples were obtained by expectoration into polypropylene tubes prior to clinical measurements. The saliva samples were weighed and then immediately frozen at -40°C until the sample collection period was completed. The samples were then lyophilized and stored at -20°C until subsequent biochemical analyses. No adverse effects associated with saliva sampling were reported.

### Acquisition of mid-IR spectra from saliva samples

Duplicate 50 μl saliva aliquots were dried at 25 Torr on 13 mm BaF windows. IR spectra were recorded using a Spectrum One FT-IR spectrometer (Perkin-Elmer, Fremont, CA) at a nominal resolution of 2 cm^-1^, with a blank BaF window employed for background measurement. 256 scan signals were averaged for each film, as we have previously described [26-28]. For the generation of the mean spectra from each group and for band integrations, the spectra were pre-processed for area normalization at the range of 1480-1750 cm^-1 ^and baseline corrected to avoid errors or artificial interference during the sample preparations and spectral acquisitions. Band integrations of relative components of each spectrum were calculated using Grams/32 AI software (Thermo Scientific). This software integrates and computes the area of selected peaks by defining two endpoints on the bottom trace of the spectrum. To make analyses consistent and reproducible, the endpoints for each band area integration were predefined to avoid artificial errors. Original absorbance spectra were also converted into second derivative spectra using the Savitzky/Golay algorithm with a 9-point window prior to multivariate statistical analysis.

### Generation of diagnostic algorithms for diabetes (sensitivity, positivity, predictive values)

We generated combined (type 1 and type 2) diagnostic algorithms, essentially as we have reported previously [19,26,27]. The diagnosis of each saliva sample was provided prior to linear discriminant analysis (LDA) calculations. Thereafter, the processed spectra were further exposed to the optimal region selection genetic algorithm, which identified a set of discrete spectral sub-regions that maximally enhance the differentiation among the various spectral subtypes. In particular, LDA was used to partition the saliva samples into disease and non-disease groups according to the discriminatory patterns in the data and into a validation set which was then used to test the accuracy of the trained algorithm. LDA assumes multivariate normality and covariance matrices of the groups are equal. Sensitivity, specificity and positive and negative predictive values were determined for the classification process through cross-validation. Approximately two thirds of the samples were designated the training set, the remaining one-third the test set, again, as we have previously described [19,26,27].

## Results

### Infrared spectral features of saliva

**Figure 1:**
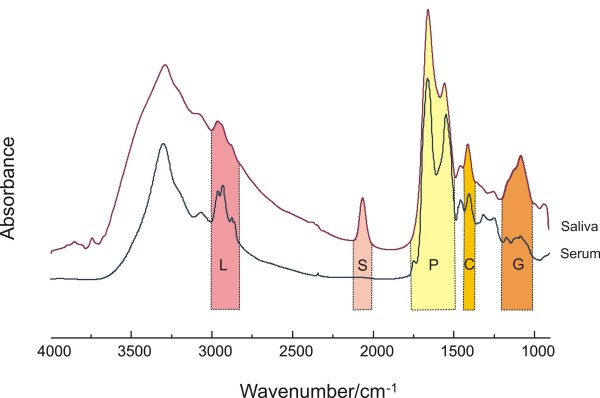
**Comparison of IR spectra obtained from films of normal human saliva and serum**. Areas marked **L, S, P **and **G **represent lipid, thiocyanate, protein and glucose, respectively.

To better understand the characteristics of infrared spectral features embedded in human saliva, we first compared saliva spectra to that of serum, as shown in Figure 1. This permitted us to correlate specific spectral regions (shaded areas) in saliva with their respective, established features in serum.

The strong bands at 2852 and 2926 cm^-1^, highlighted L (lipid), originate from the symmetric and asymmetric stretching vibrations of lipid acyl CH_2 _groups. P (protein) contains the two major prominent amide absorptions - one at 1655 cm^-1^, (arising from C = O stretching, and termed the amide I band) and another at 1546 cm^-1^, originating from N-H bending (termed the amide II band) vibrations of the peptide groups in proteins. The IR vibrational bands assigned to glucose, glycogen or sugar moieties are shown in the area highlighted in **G **(950 -1180 cm^-1^). Generally, the intensity of lipid side chains found in serum is higher than that in saliva; while the glycosylation associated sides chains of spectral vibration absorbances are more prominent in saliva. This may reflect a large contribution of AGEs and ALE's to salivary spectra.

**Figure 2:**
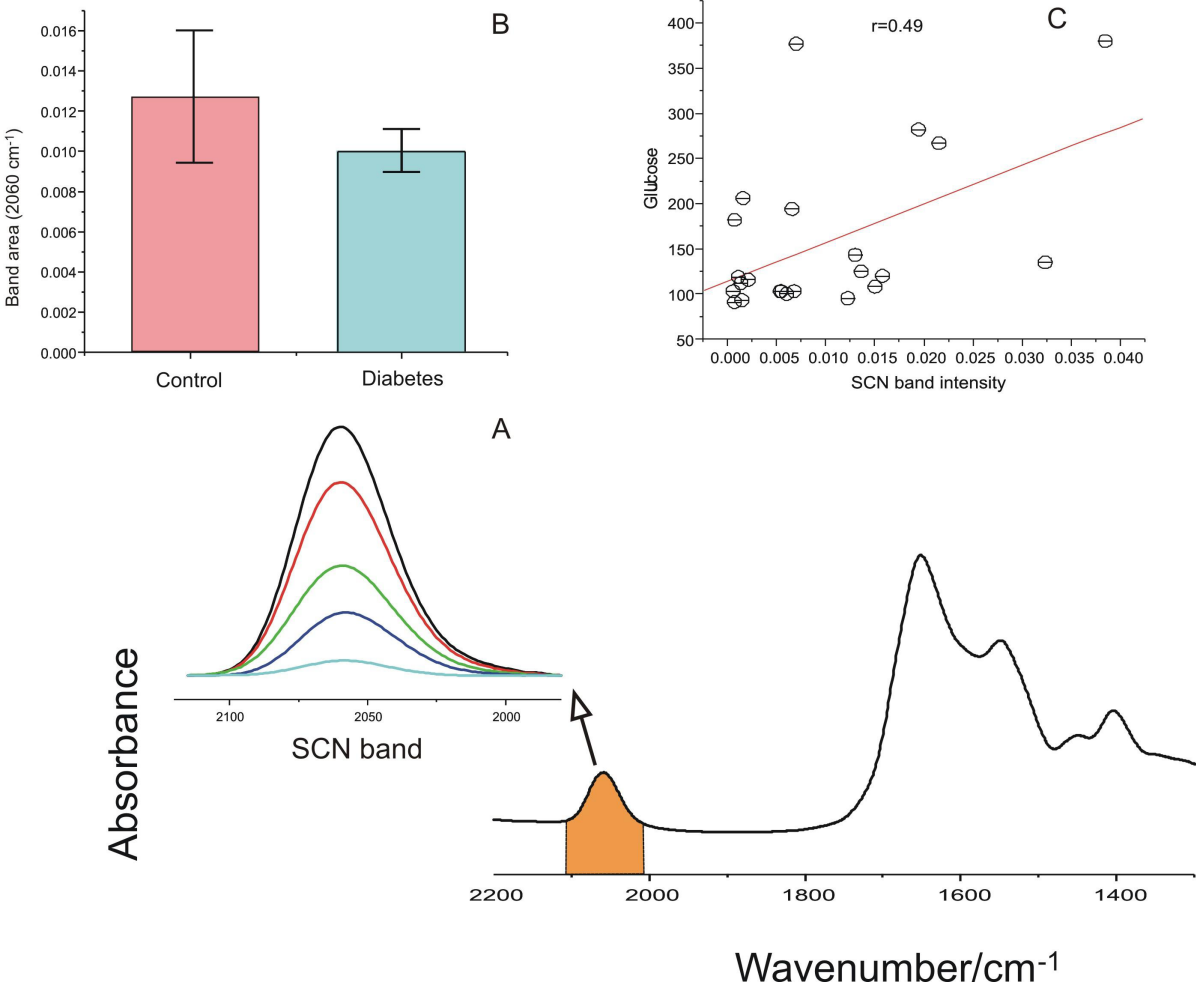
**Thiocyanate and glucose signatures in the IR spectra of saliva samples**. (A) Representative thiocyanate band intensities in the IR spectra of saliva from diabetes (n = 2) and control subjects (n = 2) chosen to highlight that clear differences in salivary thiocyanate signals are readily apparent in saliva; (B) The histogram represents the integrated area (mean, s.e. SCN^- ^content) in subjects with diabetes (red bar) and healthy controls (blue bar); and (C) the correlation plot revealing the association between SCN- band intensity and glucose concentration in the saliva of the diabetes subjects.

Our group has previously confirmed that SCN^- ^can be accurately quantitated in human saliva by IR spectroscopy because of its unique band position and lack of interference from other salivary components [29]. Indeed, the band highlighted in **S**, which derives from thiocyanate (SCN^-^) anions, is unique to saliva. In saliva, SCN^- ^is converted by salivary perioxidases to hypothiocyanite (OSCN^-^), a local antibacterial agent with high efficiency [30]. Several studies have demonstrated the thiocyanate level is closely associated with the smoking status of the subject [31]. Since this band only exists in the spectrum of saliva, we attempted to verify whether it buries any biochemical information regarding the status of diabetes. To this end, by integrating this band area from two groups, we compared the relative concentrations in both groups and correlated them with the blood glucose concentrations in the diabetic group, as shown in Figure 2. However, there is no significant difference between the band areas generated from the two groups nor there is any correlation between this band and the blood glucose concentrations. Neither was there any correlation between salivary IR spectra and circulating HbA_1_C levels (data not shown).

The IR spectrum of saliva includes specific spectral contributions from protein, lipid, glucose, and other metabolic compounds. Because of overlapping absorptions, as shown in Figure 1, IR based analytical methods which use spectral information spread across a wide spectral range are commonly employed. We used Fourier self-deconvolution to narrow effective bandwidths, enhance resolution, and increase available discriminatory data to more fully utilize the biochemical information embedded in salivary spectra.

**Figure 3:**
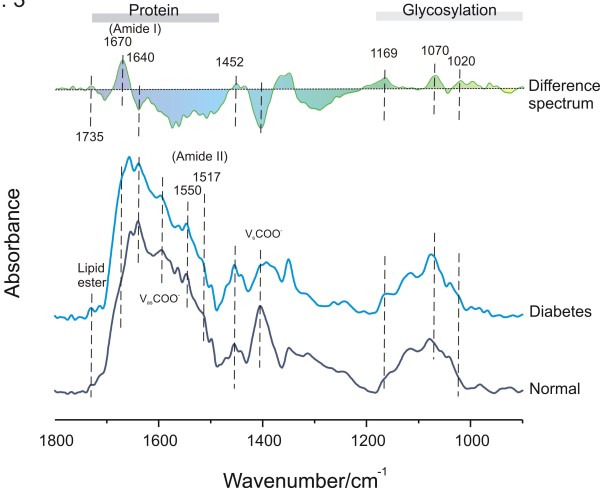
**General features of FSD-processed mean IR spectra of control and diabetes (bottom) subjects and the difference spectrum (diabetes minus control, top)**. Note: Although some non-highlighted bands exhibit pronounced differences, they are not known to convey significant meaning in terms of biological significance.

Differences in FSD-processed mean IR spectra from normal (control) and diabetic saliva are presented in Figure 3. The difference spectrum helps identify those molecular components most at variance between two groups of spectra. The altered α-helix (1640 cm^-1^) component in the amide I region is obvious in the spectrum of diabetic saliva and is related to the decreased intensity of intermolecular antiparallel β-sheets (1670 cm^-1^). The vibration of the tyrosine ring (1517 cm^-1^) in many proteins is also altered in the diabetic group. The amide II band at 1550 cm^-1^, resulting from N-H bending, was less prominent in diabetic saliva than those from normal saliva, while the lipid ester band at 1735 cm^-1 ^was more intense. This is interesting as it has been known for some time that saliva contains cholesterol levels that reflect serum concentrations[32]. Furthermore, we have previously used IR spectroscopy to quantitate cholesterol (HDL and LDL) in serum [28]. The bands marked v_s_COO^-1 ^and v_as_COO^-1^, located at 1400 and 1582 cm^-1^, are the symmetric and asymmetric carboxyl radical stretching vibrations of carboxylate groups, such as those in lactic acid or side chains of protein in saliva. Both carboxyl bands from diabetic saliva decreased compared to controls. Interestingly, several genes controlling carboxylic acid metabolism have previously been shown to be dysregulated in diabetic subjects [33]. The band at 1452 cm^-1^, originating from the bending vibration of CH_2 _group of amino acids in protein side chains, also changed in diabetic group. Another important area in the IR spectrum is the spectral range 950-1180 cm^-1 ^that originates from various C-C/C-O stretching vibrations in sugar moieties. The 1020 cm^-1 ^band is usually attributed to the C-O stretch vibration in glycogen while the bands at 1070 and 1169 cm^-1 ^can be assigned as C-O-C symmetric and asymmetric vibrations of sugar moieties and phospholipids. Obviously, therefore, the contribution of AGEs and ALE's to diabetic spectra may be large.

### Diagnostic accuracy of diabetes based on IR spectra of saliva

**Figure 4:**
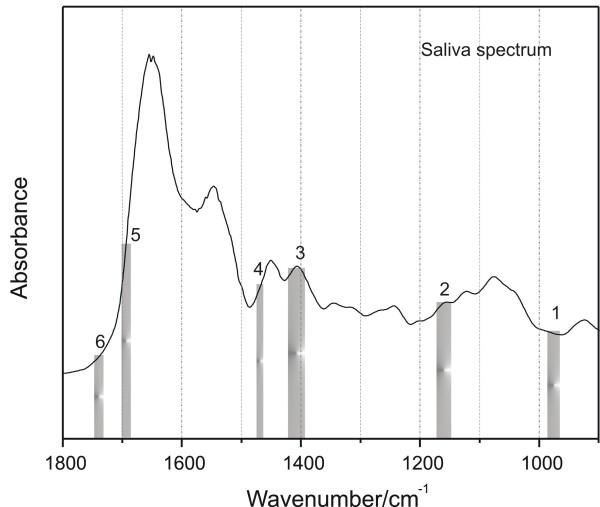
**Linear discriminant analysis of the normal and diabetic groups**. The bars identify the six spectral regions selected by the optimal regional selection algorithm that best contribute to the differentiation of normal and diabetic groups by linear discriminant analysis.

Initially, we carried out a principle component-discriminant function analysis (PC-DFA). However, the separation between the test and control groups was not ideal (data not shown). Therefore, LDA was employed to identify six spectral regions that best contribute to the differentiation of normal and diabetic groups. These spectral features are presented in Figure 4.

The sensitivity, specificity, and positive predictive value of the infrared spectra-based diagnostic algorithms for diabetes are presented in Table 2. The diagnostic sensitivity for diabetes of infrared spectroscopy was 100.0% for the training set and 90.9% for the test (validation) set; while the overall accuracy was 100% on the training set, 88.2% on the test set.

**Table 2:** Diagnostic accuracy of diabetes based on IR spectra of saliva

		n	Accuracy (%)	SP (%)^a^	PPV (%)^b^	NPV (%)^c^
	**Training Set**					

**Diabetes**	**28**	0	100.0	100.0	100.0	100.0

**Non-diabetes**	0	**8**	100.0	100.0	100.0	100.0

	**Validation Set**					

**Diabetes**	**9**	0	100.0	75.0	81.8	100.0

**Non-diabetes**	2	**6**	75.0	100.0	100.0	81.8

Overall accuracy: 100% on the training set, 88.2% on the test set. Diagnosis of diabetes was determined by linear discriminant analysis of the infrared spectra. Bold numbers are indicative of accurate classifications. Underlined numbers are indicative of inaccurate classifications. The accuracy column also represents sensitivity for diabetic patients (but not control subjects). SP = specificity; PPV = positive predictive value; NPV = negative predictive value.

## Conclusions

Early diagnosis is key to the management of diabetic complications, yet approximately 25% of Americans with diabetes (> 6 million people) are thought to be undiagnosed [34]. A contributing factor to the under-diagnosis of diabetes is a lack of simple, rapid, non-invasive, accessible, and inexpensive point-of-care diagnostic tools. To this end, we have investigated the potential of a novel, non-invasive, infrared spectroscopy-based modality to identify disease-specific alterations to the molecular profile of saliva.

Saliva has multiple potential advantages over blood testing, as recently reviewed [35]. These include the fact that saliva collection is considered non-invasive by patients; collection of saliva is safer (needle stick risk eliminated); it is convenient to collect (phlebotomists are not required); and it can be collected at home and delivered to nearby facilities [35]. For specific purposes, it may even be possible to mail saliva samples from a patient's home to the point of analysis. In the long term, it has been conceived that an increased interest in salivary diagnostics will "help catalyze a shift (in medical practice) from disease diagnosis to health surveillance" [35]. As noted earlier, saliva contains multiple components whose concentrations are altered by diabetes [13-15,17,18], some of which have strong diagnostic potential [13,15]. The use of IR spectroscopy allows the simultaneous measurement of all saliva components. Therefore, our approach differs fundamentally from prior studies that have attempted to quantify predominantly single saliva analytes, without convincing success.

On comparing the unprocessed spectra of saliva and serum it was apparent that the major differentiating spectral feature was the thiocyanate signal which, despite the complexity of saliva, was free of interfering absorptions [29]. Following Fourier self-deconvolution, the most striking difference between the spectrum of diabetic saliva and that of control were vibrations arising from sugar moieties and/or glycosylation products, such as AGEs. This is consistent with previous reports that stimulated or unstimulated salivary glucose concentrations are higher in diabetic patients than in control subjects [36,37]. These findings are also in keeping with numerous studies that have shown increased salivary AGE content on the development of diabetes complications [38] .

Also associated with diabetes was a decreased lactic acid signature. Interestingly, the role of lactate in the modulation of hormone release and responsiveness and in the control of homeostasis is being increasingly appreciated, as recently reviewed by Sola-Penna [39]. The protein profile, on the other hand, is increased in the spectra of diabetes subjects relative to healthy controls.

Diabetes is clearly multifactorial, yet previous attempts to use saliva analysis as a diagnostic tool have generally relied on the measurement of only one or two specific saliva components. Since IR spectroscopy measures the composite molecular content of saliva then, assuming molecular alterations occur during the disease process, then the chance for IR spectroscopy to distinguish various states of diabetes should be promising - in contrast to previous one-dimensional biochemical approaches. This hypothesis is supported by our LDA analyses, which consider multiple components in the saliva as the basis to designate individual spectra as healthy or diseased. Even with our relatively small number of subjects (***n ***= 61) the overall accuracy for the diagnosis of diabetes based on infrared spectroscopy was 100% for the training set, 88.2% for the test set. Such results leave us confident that an expanded subject base will improve the accuracy of diagnostic algorithms generated from IR spectra and lead to still improved sensitivities and positive predictive values.

Several of the differentiating spectral features selected by LDA have clear relevance to physiological mechanisms that underlie diabetes. Measurement of circulating glycated end products, particularly glycated hemoglobin (HbA_1c_) can be used (i) as an adjunct to glucose measurement to monitor effective control of diabetes [1,2,40] and (ii) as an independent risk factor for diabetic complications, particularly vascular complications [16,40]. While hemoglobin is generally only found in saliva at minute levels, many other glycated proteins will be present. Indeed, we show that multiple bonds with strong infrared absorptions that are present in glycated proteins are found in the saliva and represent diabetes-related structural shifts that are readily determined by infrared spectroscopy. Since multivariate analysis bares the risk of overfitting, is extremely important to clearly separate the training and validation/test set. This has been performed for our LDA and specificity and sensitivity analyses. However, it should be noted that optimal differentiating regions were identified by using the complete data set.

An IR-based approach holds several attractions for diabetic screening. Briefly, for such inexpensive, rapid, and reagent-free multi-parameter testing only small amounts of sample (50 μl saliva) are required; dried films are expected to be stable over the long-term (unlike, e.g., blood glucose); and the technology can be readily automated. It is simpler and less expensive than other high-throughput techniques, such as proteomic analysis of biofluids from diabetic subjects [41]. Furthermore, collection of saliva is non-invasive, simple, and can be performed almost anywhere, including point-of-care sites, without the need for the extensive training, for example, for phlebotomy. IR may also be useful in research into diabetes as the study of specific molecular features of the saliva of diabetics may identify diseases mechanisms, novel risk factors for diabetic complications; and novel markers of therapeutic efficacy.

In conclusion, we have assessed global, diabetes-associated alterations to saliva at the molecular and sub-molecular levels by using infrared spectroscopy and have established that infrared spectroscopy can be used to generate a complex biochemical profile of saliva and can identify several diabetes-specific spectral features. Such a tactic differs fundamentally from existing approaches that have examined individual (normally glucose) or, at best, small numbers of molecules as potential disease biomarkers. While our conclusions as to the diagnostic potential of IR are limited by the small number of subjects, further study into the potential utility of infrared spectroscopy as a diagnostic, prognostic, and research tool for diabetes seems warranted.

## Competing interests

Two of the authors DA Scott and K-Z Liu are inventors of the U.S. provisional patent application number 61/139,263. All other authors declare no current competing interests.

## Authors' contributions

SC, PM and NB carried out the clinical sections of the study and contributed to drafting the manuscript. KZL carried out the statistical analyses of infrared spectra and contributed to the drafting of the manuscript. DAS conceived the study while DAS and NB participated in the study design. NB also performed the statistical analysis of the clinical data. DAS also contributed to data analysis and drafted the manuscript. DER performed the biochemical and infrared analyses and contributed to the drafting of the manuscript. All authors read and approved the final, submitted version.
